# Teriflunomide Attenuates Demyelination and Enhances Remyelination in Organotypic Brain Slice Cultures Through Modulation of Glial Cell Dynamics

**DOI:** 10.1002/cns.70857

**Published:** 2026-04-13

**Authors:** Jessica Kronenberg, Lara‐Jasmin Schröder, Henriette Reinsberg, Thomas Skripuletz, Sandra Heckers, Florian Hansmann, Wolfgang Baumgärtner, Martin Stangel, Viktoria Gudi

**Affiliations:** ^1^ Department of Neurology Hannover Medical School Hannover Germany; ^2^ German Aerospace Center (DLR) Institute of Aerospace Medicine, Radiation Biology Köln Germany; ^3^ Center for Systems Neuroscience University of Veterinary Medicine Hannover Hannover Germany; ^4^ Institute of Pathology Hannover Medical School Hannover Germany; ^5^ Department of Pathology University of Veterinary Medicine Hannover Hannover Germany; ^6^ Institute of Veterinary Pathology Leipzig University Leipzig Germany; ^7^ Translational Medicine Neuroscience Biomedical Research, Novartis AG Basel Switzerland

**Keywords:** astrocytes, microglia, multiple sclerosis, oligodendrocytes, organotypic cerebellar slice culture, teriflunomide

## Abstract

**Objectives:**

Teriflunomide has been proven to be effective in the therapy of relapsing–remitting multiple sclerosis (RMS). In an approach to better elucidate the mode of action of teriflunomide in the central nervous system (CNS), we aimed here to clarify the role of teriflunomide on glial cells using an ex vivo demyelination model.

**Methods:**

Organotypic cerebellar slice cultures (OSC) were cultivated from 10‐day‐old mice and left to fully myelinate for another 7 days. Demyelination was induced by lysolecithin (LPC) and was studied by immunohistochemistry against myelin proteins and electron microscopy. Glial cell responses were investigated by immunohistochemistry. Intra‐glia interactions were studied using primary rodent glial cell cultures.

**Results:**

Teriflunomide treatment did not affect developmental myelination but attenuated myelin degradation induced by LPC, as assessed by myelin basic protein and myelin oligodendrocyte glycoprotein immunoreactivity. During demyelination, teriflunomide treatment was associated with reduced microglial cell density and proliferation. Partial depletion of microglia using the CSF‐1R inhibitor BLZ945 resulted in a similar preservation of myelin, supporting a functional association between microglial abundance and the extent of myelin loss in this model. Quantitative ultrastructural analysis further supported preserved myelin structures in teriflunomide‐treated slices. Spontaneous remyelination was improved and enhanced numbers of oligodendrocytes were detected following teriflunomide treatment in OSC. However, direct cytoprotective/pro‐proliferative effects of teriflunomide on oligodendroglia were not observed in primary glial cultures. There were also no indirect effects of teriflunomide‐treated microglia on oligodendrocyte progenitor cells in vitro.

**Conclusions:**

Teriflunomide exerts beneficial effects on myelin preservation and remyelination in an ex vivo demyelination model, potentially through modulation of glial cell dynamics rather than direct effects on oligodendroglial cells.

## Graphical abstract

In an organotypic cerebellar slice culture (OSC) ex vivo demyelination model, teriflunomide has been shown to positively affect myelin preservation during demyelination and enhance remyelination. This is hypothesized as being achieved by modulating the dynamics of glial cells.

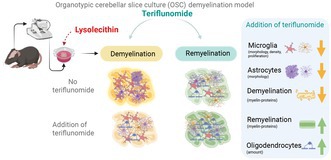

**Abbreviations**
APC: adenomatous polyposis coli
BLZ945: sotuletinib, CSF‐1R inhibitor
BrdU: bromodeoxyuridine
CNS: central nervoussystem
CSF‐1R: colony‐stimulating factor‐1 receptor
DHODH: dihydroorotate dehydrogenase
DIV: days‐in vitro
FBS: fetal bovine serum
GFAP: glial fibrillary acidic protein
IBA‐1: ionized calcium binding adaptor molecule 1
LPC: lysolecithin
LPS: lipopolysaccharide
MBP: myelin basic protein
MS: multiple sclerosis
NGS: normal goat serum
OLIG‐2: oligodendrocyte transcription factor 2
OPC: oligodendrocyte precursor cells
OSC: organotypic brain slice culture, or organotypic cerebellar slice culture
PBS: phosphate‐buffered saline
PFA: paraformaldehyde
PLL: poly‐l‐lysine
ROI: region of interest
RRMS: relapsing–remitting multiple sclerosis
SEM: standard error of the mean
TF: teriflunomide
TMEV: theiler's murine encephalomyelitis virus

## 1 Introduction

Teriflunomide (Aubagio) has been approved since August 2012 in the EU as a treatment for relapsing–remitting multiple sclerosis (RRMS). Its primary mode of action consists of reversible and noncompetitive inhibition of the mitochondrial enzyme dihydroorotate dehydrogenase (DHODH) [1, 2, 3]. The enzyme DHODH is required for de novo pyrimidine synthesis of proliferating lymphocytes. Thereby teriflunomide reduces the number of activated peripheral T and B lymphocytes, which can infiltrate into the central nervous system (CNS). Resting lymphocytes rely on DHODH independent salvage pathways for pyrimidine synthesis and thus remain unaffected by teriflunomide treatment [4, 5]. Moreover, another DHODH independent mechanism, the inhibition of cytokine release that was not reversed by addition of uridine, has been shown [6]. Although teriflunomide has only a low blood–brain‐barrier penetration, approximately 1%–2% of the serum concentration is found in the brain parenchyma, which corresponds to a concentration of at least 2.5–4.1 μM [7]. This implies that cells of the CNS, such as microglia or oligodendrocytes, may be exposed to and modulated by teriflunomide treatment. Indeed, the previous studies by Gottle et al. confirm this assumption [8, 9]. In our former study, we could demonstrate that microglia treated with teriflunomide showed an increased expression of the anti‐inflammatory interleukin‐10 (IL‐10) after lipopolysaccharide (LPS) treatment and a reduced proliferation in mixed glial cell cultures [10]. In addition, teriflunomide decreased the release of several pro‐inflammatory cytokines from activated monocytes in a DHODH‐independent mechanism [6]. These results suggest that teriflunomide may modulate de‐ or remyelination due to its anti‐proliferative and anti‐inflammatory effects on microglia or by direct stimulation of OPC maturation.

Thus, in this study, we investigated the effect of teriflunomide treatment on glial cells during developmental myelination and during de‐ and remyelination induced by lysolecithin in organotypic brain slice cultures (OSC), a widely used system to study these processes in vitro with the advantage of preserving the complex cytoarchitecture of the CNS and allowing the interactions between cells to reflect a situation close to the in vivo environment [11, 12, 13].

## 2 Materials and Methods

### 2.1 Organotypic Brain Slice Culture

Organotypic brain slice cultures (OSC) were prepared as previously described [14]. Hence, 350 μm thick parasagittal slices of the cerebellum from postnatal 10‐day‐old (P10) C57BL/6 mice were cut using a vibratome (Leica VT1000 S Vibrating blade microtome). Slices were cultured on Millicell‐CM culture inserts (Millipore, Germany) in medium containing 50% minimum essential medium (Invitrogen, Carlsbad, USA), 25% hank's balanced salt solution (Lonza, Beligum), 25% horse serum (Invitrogen, CA), 1% penicillin/streptomycin, 6.5 mg/mL glucose (Invitrogen, USA), 2 mM L‐glutamine (Thermo Fisher Scientific, USA) for a maximum of 14 days at 37°C and 5% CO_2_. Medium was changed every 2–3 days.

### 2.2 Pharmacological Treatment of Organotypic Brain Slice Culture

**FIGURE 1:**
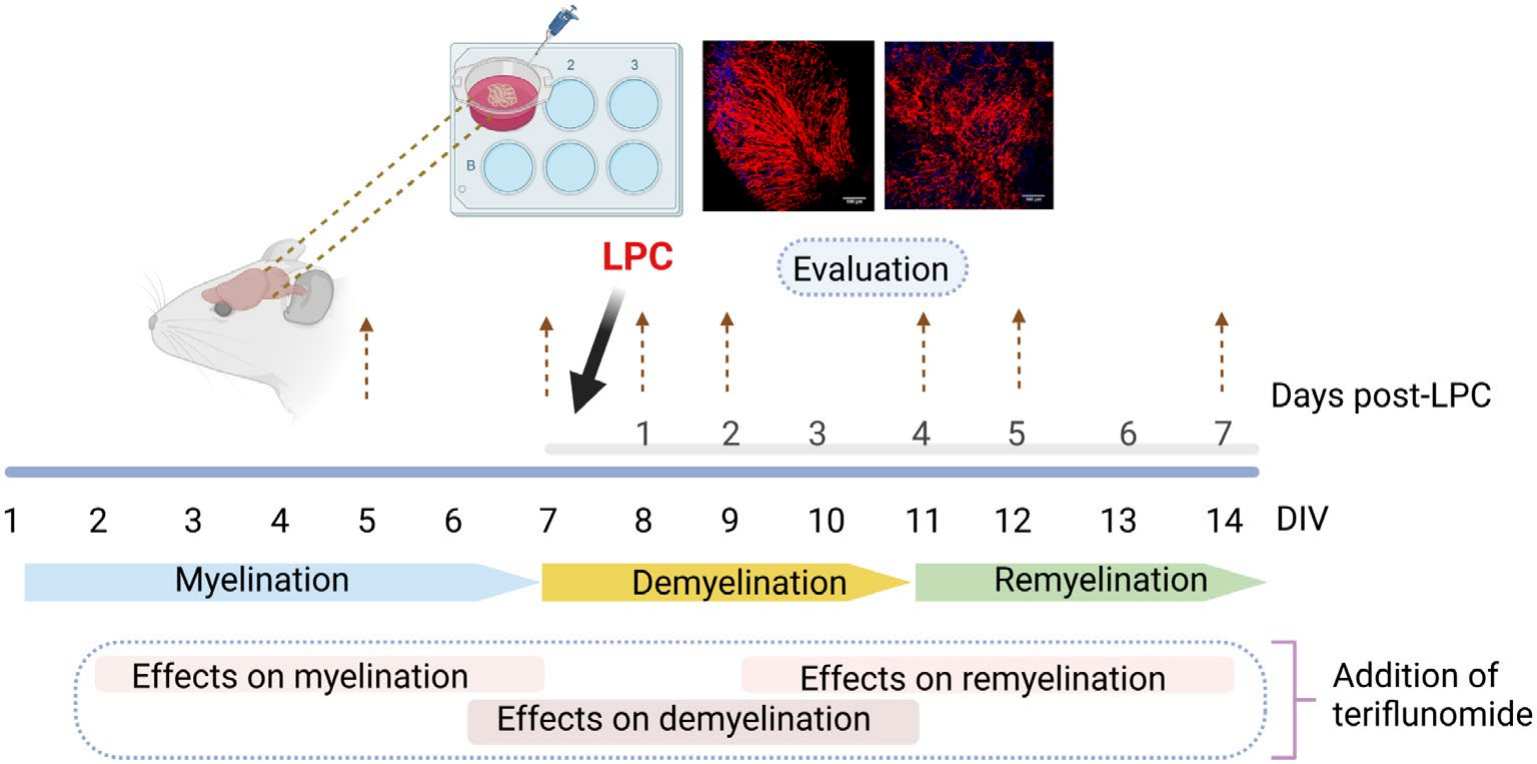
Pharmacological treatment of OSC. Experimental setup: OSC from 10 days old mice were prepared and exposed to 3, 10, or 25 μM teriflunomide at indicated time frames of myelination, de‐ or remyelination until slice fixation (dashed arrows). DIV, days‐in vitro; LPC, lysolecithin. Created with bioRender.

Teriflunomide was provided by Genzyme (Waltham, USA) and reconstituted in dimethyl sulfoxide (DMSO, Sigma‐Aldrich, USA). The teriflunomide stock (10 mM) was directly diluted in culture medium and changed every 2–3 days. The experimental setup and incubation times of the OSC are shown in Figure 1. For the myelin maintenance study, cultures were treated for five subsequent days with 25 μM of teriflunomide (2–7 days‐in vitro (DIV)). For the de‐ and remyelination study, cultures were demyelinated with lysolecithin (0.5 mg/mL; LPC) for 15–17 h after 7 DIV as previously described [15]. To determine the effect of teriflunomide on demyelination, slices were incubated with 3, 10 or 25 μM from 6 DIV until 8, 9 or 11 DIV. For the microglia inhibition studies, the OSC were simultaneously treated with the commercially available inhibitor BLZ945 (MCE, MedChemExpress, USA), also known as sotuletinib, which inhibits the receptor for colony‐stimulating factor 1 (CSF‐1R) and leads to the depletion of microglia. For the remyelination study, cultures were treated with 25 μM of teriflunomide from 9 DIV until 12 or 14 DIV.

### 2.3 Whole Mount Immunohistochemistry

Slices were fixed for 1–2 h in 4% paraformaldehyde (PFA), washed with PBS and permeabilized for 1–2 h in 0.6% Triton X‐100 (Serva, Heidelberg, Germany). After blocking with 0.3% Triton X‐100 + 5% normal goat serum (NGS) for at least 4 h, the slices were incubated with primary antibody diluted in 0.3% Triton X‐100 for 48 h by 4°C. After washing three times with PBS, slices were incubated with secondary antibody diluted in 0.3% Triton X‐100 overnight by 4°C. Slices were washed three times with PBS and finally mounted on glass slides.

The following primary antibodies were used: oligodendrocyte transcription factor 2 (OLIG2, polyclonal rabbit, 1:500, Millipore), Adenomatous polyposis coli (APC, mouse IgG2b, 1:200, Calbiochem), myelin basic protein (MBP, mouse IgG2b, 1:500, Biolegend, USA), myelin oligodendrocyte glycoprotein (MOG)‐specific hybridoma supernatant (generated from hybridoma cells provided by Christopher Linington, University of Glasgow, UK, 1:2), Glial fibrillary acidic protein (GFAP, polyclonal rabbit, 1:200, Millipore, USA), Ionized calcium binding adaptor molecule 1 (IBA‐1, polyclonal rabbit, 1:200, Wako, Germany), Ki‐67 (mouse IgG1, 1:100, BD Pharmingen, San Jose, USA). Secondary antibodies were goat anti‐mouse Alexa‐550 and goat anti‐rabbit Alexa‐488 (all from Invitrogen, Thermo Fisher Scientific). In organotypic cerebellar slice cultures, myelin integrity was primarily assessed using immunostaining for myelin oligodendrocyte glycoprotein (MOG). MOG (type 1 transmembrane protein located on the outer side of myelin sheaths) was predominantly selected as a sensitive marker for mature myelin in the murine cerebellar white matter [16]. Previous work from our group demonstrated that, following LPC‐induced demyelination, MOG immunoreactivity more accurately reflects preserved myelin integrity, whereas MBP, which is found on the cytoplasmic side and plays a role in the compaction and stabilization of myelin leaflets, may label new‐formed myelin (relatively early expressed) but also myelin remnants [15, 17]. MBP and MOG staining was quantified based on binarized images per 20× objective image using a consistent threshold in ImageJ/Fiji (Fiji, U.S. National Institutes of Health, Maryland). Cell counts (OLIG2, IBA‐1, APC, Ki‐67) were performed on native immunofluorescent images from three individual images per slice in a blinded manner.

In this study, “*n*” refers to the number of animals. Slices were derived from multiple animals, with slices from 3 to 8 independent mice per condition in each given experiment or staining. Importantly, per each animal, 2 slices were imaged and/or analyzed in every corresponding readout. In detail, for each slice, three individual images were analyzed and averaged prior to statistical evaluation.

### 2.4 Measurement of Nitric Oxide

The stable reaction product of nitric oxide (NO), nitrite, was measured using the colourimetric Griess assay to evaluate NO production as described by Schröder et al. [15]. In brief, supernatants from the culture slices were gathered per well, purified through precipitation using 80% v/v acetonitrile, and subjected to centrifugation at 16,000 *g* for 15 min at a temperature of 4°C. The resulting pellets were left to dry at room temperature (RT). Upon rehydrating the sample to 10% of its original volume, it was combined with Griess reagent in a 1:1 ratio and incubated at room temperature for 20 min. Absorbance at 540 nm was measured using a reference wavelength of 620 nm. The absorbance at 540 nm was measured using 620 nm as a reference wavelength. Nitrite concentrations were determined using a standard curve generated in‐house, following the manufacturer's protocol (provided by Promega, Wisconsin, USA), and the values were adjusted to the mean of the untreated wild‐type controls.

### 2.5 Mixed Glia Cell Cultures

For preparation of primary mixed glial cell cultures, neonatal Sprague–Dawley rats P0‐P3 were used as previously described [18]. After freeing brains from meninges, choroid plexus and brain stem, they were minced and further enzymatically dissociated with 0.1% trypsin (Biochrom, Germany) and 0.25% DNase (Roche, Germany). The cells were then plated into culture flasks pre‐coated with poly‐l‐lysine (PLL; Sigma‐Aldrich, Germany), filled with Dulbecco's Modified Eagle Medium (DMEM; Life Technologies, USA) supplemented with 1% penicillin/streptomycin (Sigma‐Aldrich Hamburg, Germany) and 10% fetal bovine serum (FBS; Biochrom, Germany). Until use, cultures were kept at 37°C and 5% CO_2_.

After 7 days, microglia were isolated from the mixed glial cells by shaking at 37°C for 45 min at 180 rpm on an orbital shaker (Edmund Bühler, Germany). Afterwards 300.000 microglial cells were seeded on 12 well plates (Sarstedt, Germany). Microglia were incubated overnight at 37°C, 5% CO_2_ and, on the following day, pretreated with 3, 10, or 25 μM teriflunomide (stock: 10 mM, dissolved in DMSO) for 16 h followed by further stimulation with Interleukin‐4 (IL −4, 20 ng/mL; Peprotech, Germany) for 10 h. After washing with phosphate‐buffered saline (PBS), medium was changed to serum‐free culture medium for a further 16 h; microglial supernatants were then harvested and kept at −80°C until use.

Oligodendrocytes were isolated by shaking at 160 rpm for 16–20 h. Supernatants were collected, centrifuged, and cells were then transferred into an uncoated flask for 30 min at 37°C to reduce contamination of astrocytes and microglia. 80.000 cells were plated on PLL coated 12 mm glass coverslips and cultured in proliferation or differentiation medium for 24 h. Proliferation medium consisted of KnockOut DMEM/F‐12 supplemented with GlutaMAX, StemPro supplement, EGF, human FGF, PDGF‐AA (all from Thermo Fisher Scientific, Germany). Neurobasal medium supplemented with GlutaMAX, B‐27 supplement (all from Thermo Fisher Scientific, Germany), and 30 ng/mL T3 (Sigma‐Aldrich, Germany) was used for oligodendrocyte differentiation [19]. To collect astrocyte supernatants, glial cultures were incubated with cytosine arabinoside (Ara‐C; 8 μM; Sigma Aldrich, USA) for 72 h. Astrocytes were then seeded into 6 well plates (Sarstedt, Germany), and after reaching confluency, astrocytes were incubated for 16 h with DMEM without serum. Cell culture supernatants were harvested and kept at −80°C until use.

### 2.6 Treatment of Oligodendrocytes With Teriflunomide, Microglia or Astrocyte Conditioned Media

To evaluate the direct effect of teriflunomide on oligodendrocytes, cells were plated and allowed to proliferate or differentiate in normal culture medium for 24 h. Oligodendrocytes were then directly incubated with 3, 10, or 25 μM teriflunomide in culture medium for another 48 h.

To investigate whether supernatant from untreated astrocytes affects the impact of teriflunomide on OPC, cells were incubated with a ratio of 1:3 of defined culture medium supplemented with untreated astrocyte supernatants devoid of growth factors with the addition of 3, 10, or 25 μM teriflunomide for a further 48 h. To determine if microglia treated with teriflunomide with/without the indicated stimulus of IL‐4 influence differentiation or proliferation of OPC, cells were incubated with a ratio of 1:3 of defined culture media supplemented with pretreated microglia supernatants devoid of growth factors for another 48 h.

Cells were then fixed and stained as described in the following. To determine the differentiation index of mature oligodendrocytes to OPC, primary cells were incubated with anti‐A2B5 (hybridoma supernatant, clone 105, European Collection of Cell Cultures) and anti‐galactocereboside (GalC, hybridoma supernatant, clone IC‐07, European Collection of Cell Cultures) supernatants for 30 min at 37°C. After fixation with 4% PFA, cells were incubated with the secondary antibodies AlexaFluor 488 goat anti‐mouse IgG3 and AlexaFluor 555 goat anti‐mouse IgMμ 1:500 (Thermo Fisher Scientific, USA).

To investigate the percentage of proliferating OPC, cells were incubated for 3 h with 10 μM Bromodeoxyuridine (BrdU, Roche, USA). Cultures were washed with PBS and incubated with anti‐A2B5 supernatant. After fixation with 4% PFA, cells were permeabilized with methanol at −20°C and DNA was denaturized with 2 M HCl at 37°C (Roth, Germany). Cells were then neutralized with 0.1 M borate buffer pH 8.5 and stained with anti‐BrdU 1:100 (BrdU, Roche, USA) and incubated with secondary antibodies AlexaFluor 555 goat anti‐mouse IgMμ and AlexaFluor 488 goat anti‐mouse IgG 1:500 (Thermo Fisher Scientific, USA).

### 2.7 Electron Microscopy

For electron microscopy (EM), brain slices were fixed with 0.25% glutaraldehyde in 1% sodium cacodylate buffer, post‐fixed in 1% osmium tetroxide, dehydrated in a graded series of alcohol, and embedded in epoxy resin (EPON 812, Serva, Germany). Coronal semithin sections (0.5–1 μm) were cut and stained with toluidine blue. For each block, semithin sections were scanned to select the region of interest (ROI). Then, blocks were trimmed, and from each ROI, 70 nm ultrathin sections were generated. The contrast enhancement was performed using uranyl acetate (Merck KGaA, Germany) and lead citrate. For visualization and analysis, a Zeiss EM10C (Carl Zeiss, Germany) transmission electron microscope was applied.

Transmission electron microscopy (TEM) images were analyzed using ImageJ/Fiji. For each experimental group, images were acquired from comparable regions of the cerebellar white matter. Axons were classified as myelinated or unmyelinated based on the presence of a clearly defined compact myelin sheath. Only axons with a clearly identifiable cross‐sectional profile were included. The total number of axons and the number of myelinated axons were manually counted in a blinded manner, and the proportion of myelinated axons was calculated per image. For exploratory g‐ratio analysis, only axons displaying a near‐circular cross‐section and continuous myelin sheath were selected. Axonal area and total fiber area were manually delineated, and g‐ratios were calculated as the square root of the ratio between axonal area and total fiber area.

### 2.8 Statistical Analysis

All experiments were performed at least four times. GraphPad Prism version 5.02 was used for statistical analysis (GraphPad Software Inc., USA). Data distribution was tested for normality (e.g., Shapiro–Wilk test). As data did not consistently follow a normal distribution, the non‐parametric Kruskal–Wallis test followed by Dunn's post hoc test was applied. Values are presented as arithmetic means ± standard error of the mean (SEM). *p* < 0.05 was considered to indicate a statistically significant difference.

## 3 Results

### 3.1 Teriflunomide Did Not Influence the Developmental Myelination in OSC

**FIGURE 2:**
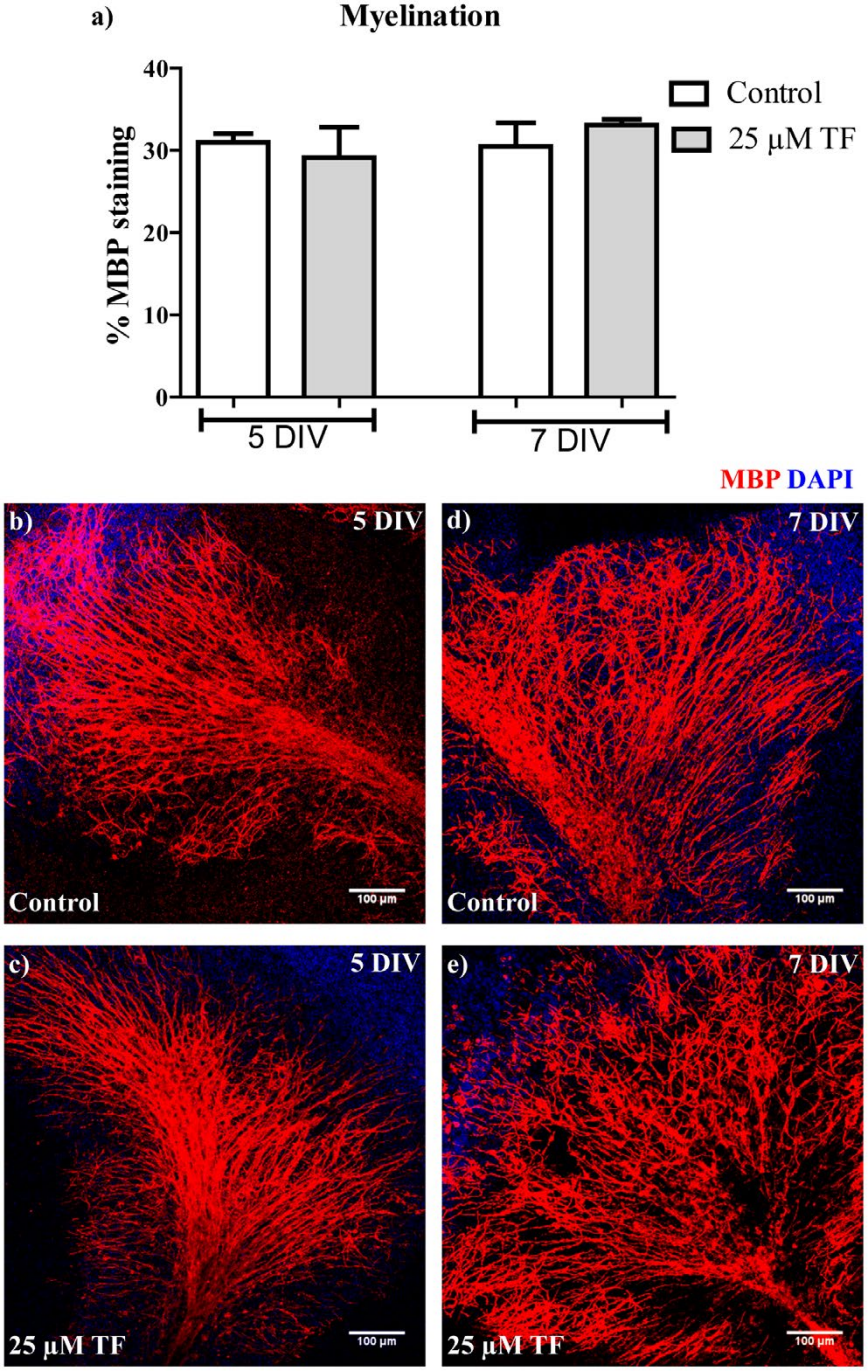
Effects of teriflunomide on myelination of OSC. OSC were treated with 25 μM teriflunomide from Day 2 (2 DIV) until fixation to determine its effect on the developmental myelination. Quantification of MBP amounts showed no effect of 25 μM teriflunomide treatment (a). Representative images of slices at 5 DIV and 7 DIV stained for MBP (b–e). Data are represented as mean ± SEM (*n* = 4). Statistical analysis was performed using Kruskal–Wallis test followed by Dunns post hoc tests. *Scale bar 100 μM*. DIV, days‐in‐vitro; TF, teriflunomide.

To investigate a possible effect of teriflunomide on developmental myelination, OSC were treated for three or five subsequent days with 25 μM teriflunomide, starting at day two (2 DIV). Slices were then analyzed immunohistochemically by MBP staining at 5 DIV and 7 DIV (Figure 2). As shown by MBP staining, axons in cultured cerebellar slices become myelinated by Days 17–21 (5–7 DIV) (Figure 2). Teriflunomide treatment did not affect MBP expression as compared to control slices, suggesting that teriflunomide might not influence developmental OSC myelination.

### 3.2 Teriflunomide Could Prevent Demyelination in OSC

**FIGURE 3:**
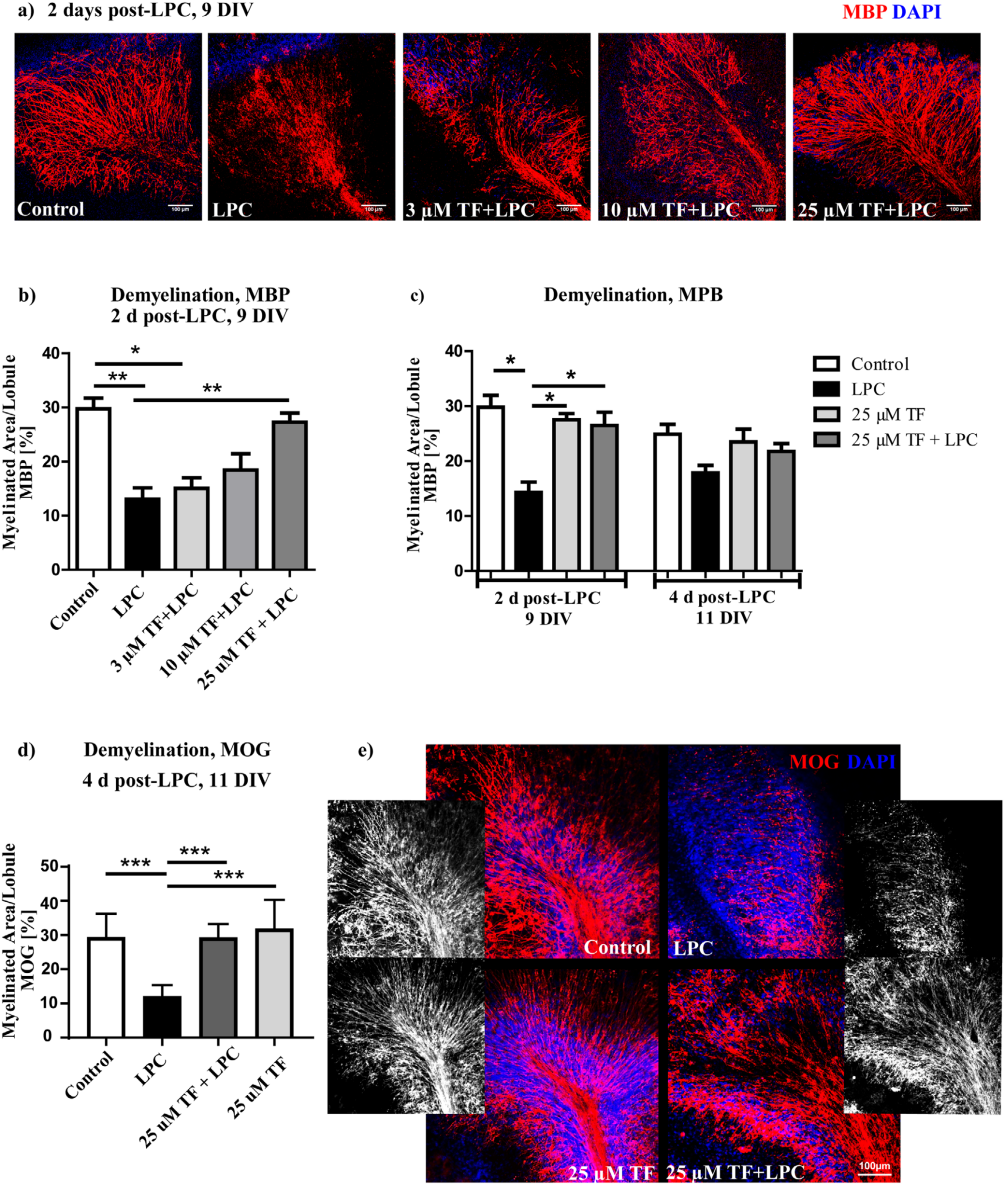
Effects of teriflunomide on demyelination of OSC. OSC were treated with 3, 10, or 25 μM teriflunomide starting at 6 DIV until fixation to evaluate its effect on demyelination. Representative images of slices at 2 days post‐lysolecithin stained for MBP and treated with different teriflunomide concentrations (a). Lower concentrations of teriflunomide had no significant effect on demyelination (b). Two days after LPC, the amount of MBP remained increased in slices treated with LPC and 25 μM teriflunomide compared to slices that received only LPC. Four days after LPC treatment, renewed expression of the myelin protein MBP was observed in LPC‐treated slices (c), whereas the myelin protein MOG was only marginally expressed at this time (d). Representative images of sections 4 days after lysolecithin stained for MOG myelin protein are shown in (e). Data are represented as mean ± SEM (*n* = 6–10). Statistical analysis was performed using Kruskal–Wallis test followed by Dunns post hoc tests (*p < 0.05; **p < 0.01). Scale bar 100 μM. DIV, days‐in‐vitro; LPC, Lysolecithin; TF, teriflunomide.

**FIGURE 4:**
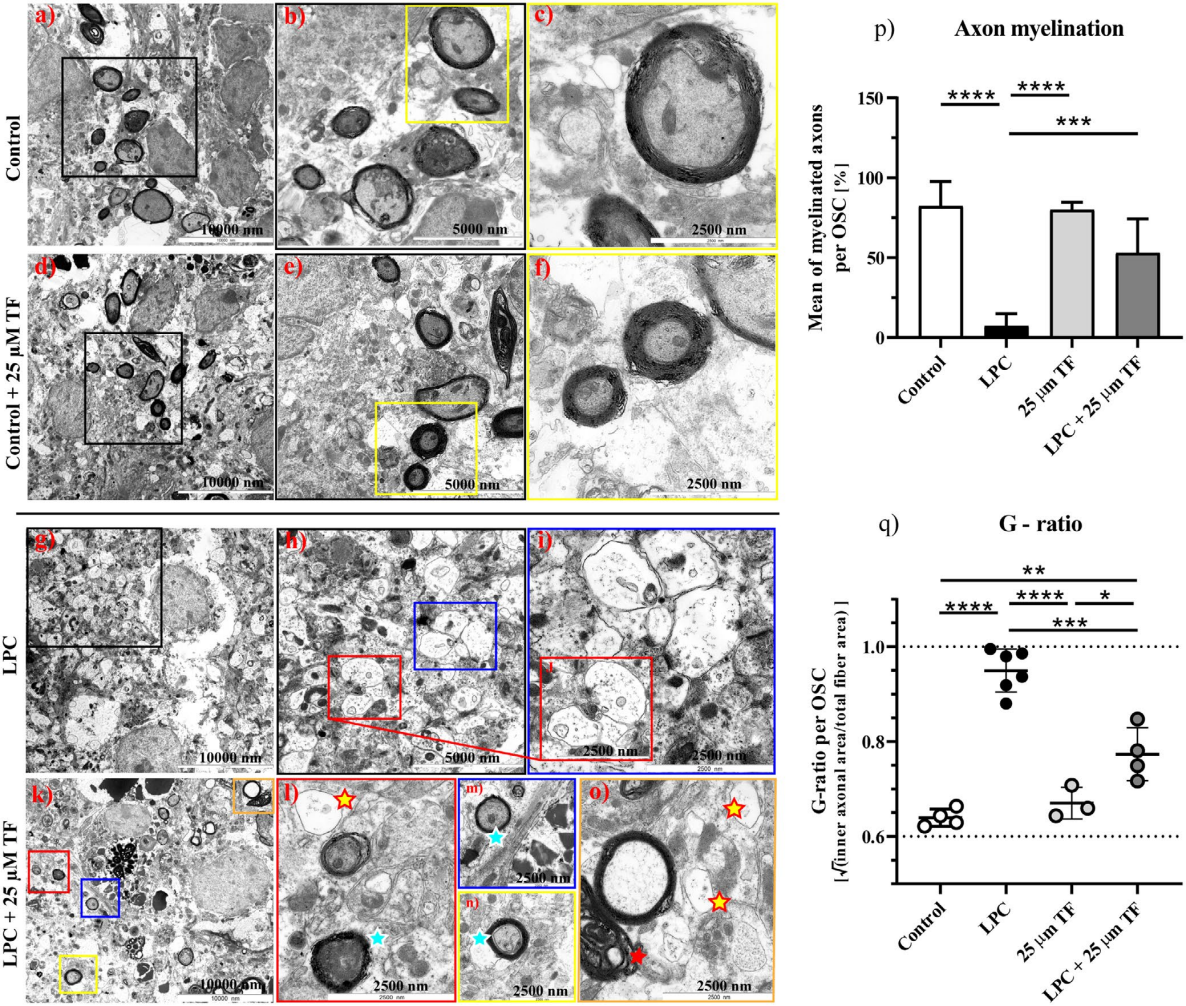
Ultrastructural analysis of myelin structures during demyelination. Representative electron microscopical images of control OSC slices (a‐c, different magnifications) and slices treated with 25 μM teriflunomide (d‐f, different magnifications) starting at 6 DIV until fixation (9 DIV) showed intact, dark, ring‐shaped myelin sheaths surrounding axons. Please note, the developmental myelination in a such artificial OSC system seems to be impaired, not so intensive, or infallible like in an intact organism since only moderate numbers of axons showed compact myelin structures and correctly folded myelin sheaths. After 2 days post‐LPC (9 DIV) demyelination occurred in OSC and most of axons became demyelinated (g–i) some of them showed an intramyelinic vacuolization (not shown). A simultaneous treatment with 25 μM teriflunomide (k–o) could extenuate demyelination and preserve myelin by numerous axons (blue asterisks), however, some axons still showed an irregular separation of myelin lamellae (red asterisk) or were not myelinated (yellow asterisk). Higher magnifications are shown in shaded frames. Scale bar 10,000 nm (a, d, g, k), Scale bar 5000 nm (b, e, h), Scale bar 2500 nm (c, f, i, j, l–o). DIV, days‐in vitro; LPC, lysolecithin; TF, teriflunomide. Quantification of myelinated axons based on TEM images (p, q). Data represent the proportion of myelinated axons per 3–5 images per animal and condition in 1.2 × 1.2 μm space. G‐ratio analysis was performed in an exploratory manner on same pictures using ImageJ. Here, *n* = 4 control, *n* = 6 LPC, *n* = 4 LPC + 25 μM and *n* = 3 TF animals were tested with each 2 slices per animal. Statistical analysis was performed using Kruskal–Wallis test followed by Dunns post hoc tests (***p < 0.001, ****p < 0.0001).

It has been shown that LPC causes a focal demyelination when applied to OSC [11]. After 7 DIV of culturing, to allow the onset of natural developmental myelination, slices were treated with 0.5 mg/mL LPC for 15–17 h. Two days post‐LPC (9 DIV) the amount of the myelin marker MBP was significantly decreased compared to the control (Figure 3), thus we indicated this time point as “severe demyelination”. Application of 25 μM teriflunomide, starting 1 day before LPC treatment until fixation at 9 DIV, prevented an LPC dependent degradation of myelin since the amount of MBP (Figure 3a,c) did not significantly differ between LPC plus teriflunomide‐treated and untreated control slices. However, when slices were incubated with 3 or 10 μM teriflunomide (Figure 3a,b), this effect was abolished suggesting a dose dependent effect of teriflunomide. Slightly increased re‐expression of MBP compared to Day 9 (9 DIV) was observed in LPC‐treated slices 4 days after LPC treatment (11 DIV). When analyzing immunohistochemical staining against the myelin oligodendrocyte glycoprotein (MOG), we still observed a significant decrease in MOG expression at Day 4 (11 DIV) in LPC‐treated sections (Figure 3d,e), probably due to a different spatial localisation of these myelin proteins, characteristic for compact myelin formation. Thus, we indicated 11 DIV as a point of “late demyelination”. A treatment with 25 μM teriflunomide almost completely abolished this phenomenon and the amount of MBP and MOG on 11 DIV remained here unchanged and comparable to the control (Figure 3a,c–e). To support these findings and to elucidate whether the myelin was preserved due to teriflunomide treatment, the ultrastructural composition of myelin were studied and a qualitative electron microscopy analysis of control, 25 μM teriflunomide, LPC, and LPC plus 25 μM teriflunomide treated OSC was performed. In untreated and teriflunomide‐only treated control slices, a limited number of axons displayed compact myelin sheaths, consistent with the incomplete developmental myelination typically observed in organotypic slice cultures (Figure 4a–f). LPC treatment resulted in significant tissue rearrangement and a marked loss of intact myelin structures (Figure 4g–j). Few myelin sheaths remained and displayed dilatation or/and irregular separation of myelin lamellae typical for demyelination. In the slices exposed to LPC plus teriflunomide, numerous axons still showed intact myelin structures (Figure 4k–o), although we could also observe some demyelinated axons or axons with vacuolized and split myelin sheaths in the LPC plus teriflunomide‐treated slices. A quantitative analysis of TEM images revealed a marked reduction in the proportion of myelinated axons following LPC treatment (7.2%) compared to controls (82.3%) (Figure 4p) as expected. Co‐treatment with teriflunomide (52.9%) partially preserved the proportion of myelinated axons compared to LPC‐treated slices. Exploratory g‐ratio analysis revealed consistent differences of myelination between experimental groups: While control and TF‐treated OSC displayed g‐ratios of 0.64 and 0.67 respectively, LPC‐treated slices had a g‐ratio of 0.95 on average. Similarly to the percentage of myelinated axons, exploratory g‐ratio of 0.77 in LPC + TF‐treated slices indicates certain myelin preservation, consistent with heterogeneous myelin integrity (Figure 4q). Moreover, we could observe only a mild tissue rearrangement compared to the control group (Figure 4), suggesting that teriflunomide could indeed ameliorate LPC‐induced demyelination in the OSC.

### 3.3 Teriflunomide Administration Did Not Affect Oligodendrocyte Dynamics During Demyelination

**FIGURE 5:**
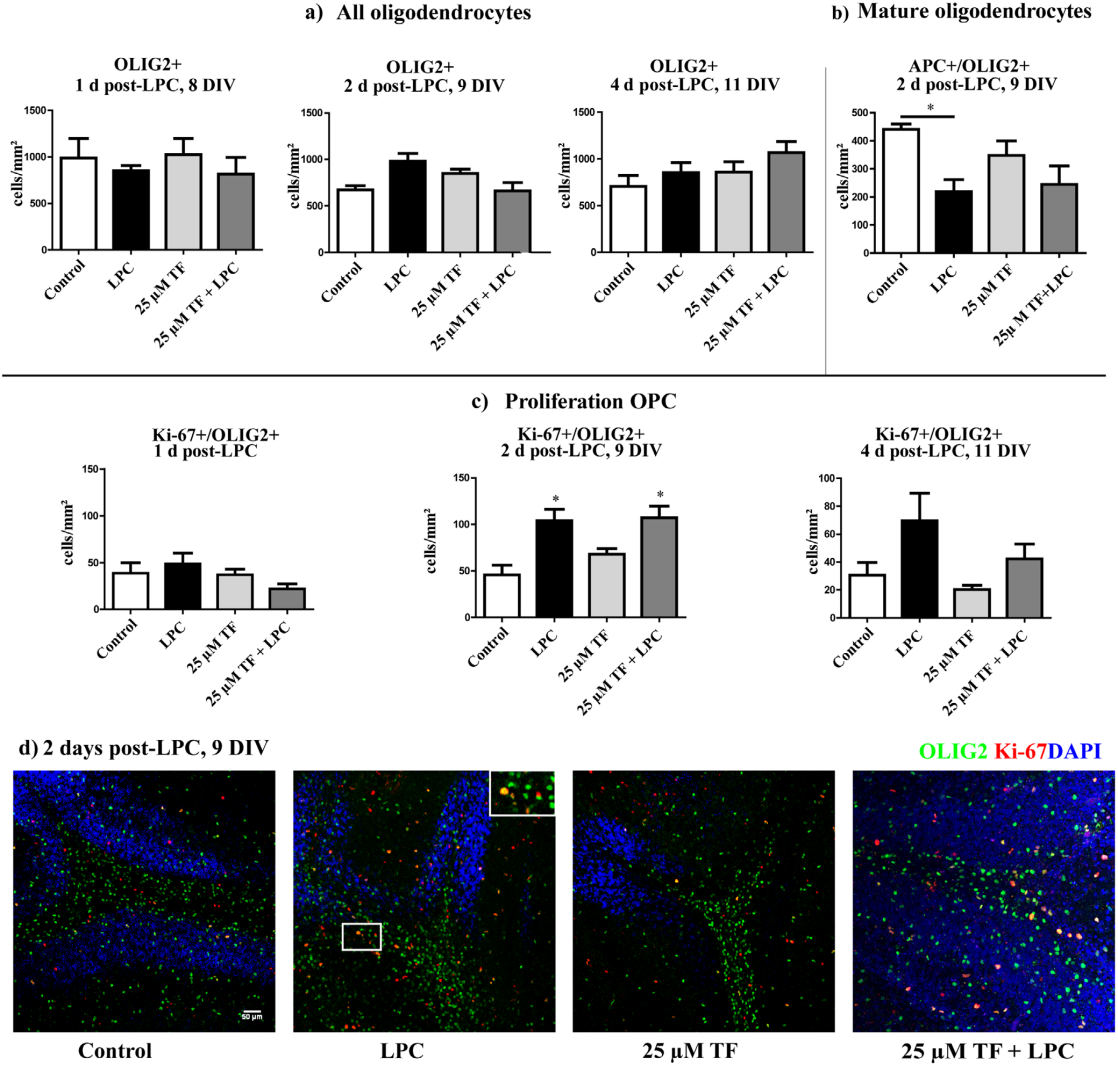
Effects of teriflunomide on oligodendrocytes during demyelination. OSC were treated with 25 μM teriflunomide starting at 6 DIV until fixation. Absolute numbers of OLIG2 positive (all oligodendroglial cells and stages) were counted in white matter after one (8 DIV), two (9 DIV), and four (11 DIV) days post‐LPC (a). Two days post‐LPC (9 DIV) the number of mature oligodendrocytes (APC/OLIG2+) were downregulated (b). Numbers of OLIG2/Ki‐67 double positive cells (proliferating young oligodendrocytes) (c) were significantly increased during demyelination, after 2 days post‐LPC, in both, LPC alone or in LPC + 25 μM teriflunomide treated slices. Representative images of slices at 2 days post‐LPC (9 DIV) treatment stained against OLIG2 and Ki‐67 (d) demonstrated effects of 25 μM teriflunomide on oligodendrocytes and their proliferation during demyelination. Data are represented as mean ± SEM (*n* = 5–8). Statistical analysis was performed using Kruskal‐Wallis test followed by Dunns post hoc tests (*p < 0.05). Scale bar 100 μM. DIV, days‐in vitro; LPC, lysolecithin; TF, teriflunomide.

The total number of OLIG2‐positive cells, labeling all stages of the oligodendrocyte lineage in the cerebellar white matter, did not significantly differ between experimental groups at one (8 DIV), two (9 DIV), or four (11 DIV) days following LPC treatment (Figure 5a).

In contrast, the number of APC+/OLIG2+ double‐positive cells, representing mature oligodendrocytes, was significantly reduced 2 days after LPC administration (9 DIV) compared to control conditions (Figure 5b), indicating LPC‐induced oligodendroglial damage.

To evaluate proliferation of immature oligodendrocytes, a double staining for OLIG2 (marker for oligodendrocytes in all stages) and Ki‐67 (proliferation marker) was performed at Day 1, 2, and 4 post‐LPC treatment (8, 9, 11 DIV). In the white matter, the number of proliferating oligodendrocytes increased after 2 days post‐LPC (9 DIV) significantly in both LPC and LPC plus teriflunomide‐treated slices (Figure 5c,d). On Day 4 (11 DIV), the numbers of OLIG2/Ki‐67 double‐positive cells in these groups were still slightly but not significantly increased as compared to control (untreated and only teriflunomide treated) slices (Figure 5c). In the gray matter, the number of proliferating oligodendrocytes was unaffected by LPC or teriflunomide treatment (25 μM) at all time points studied (Data not shown).

### 3.4 Teriflunomide‐ Mediated Preservation of Myelin During Demyelination Is Accompanied by Altered Microglia Response

**FIGURE 6:**
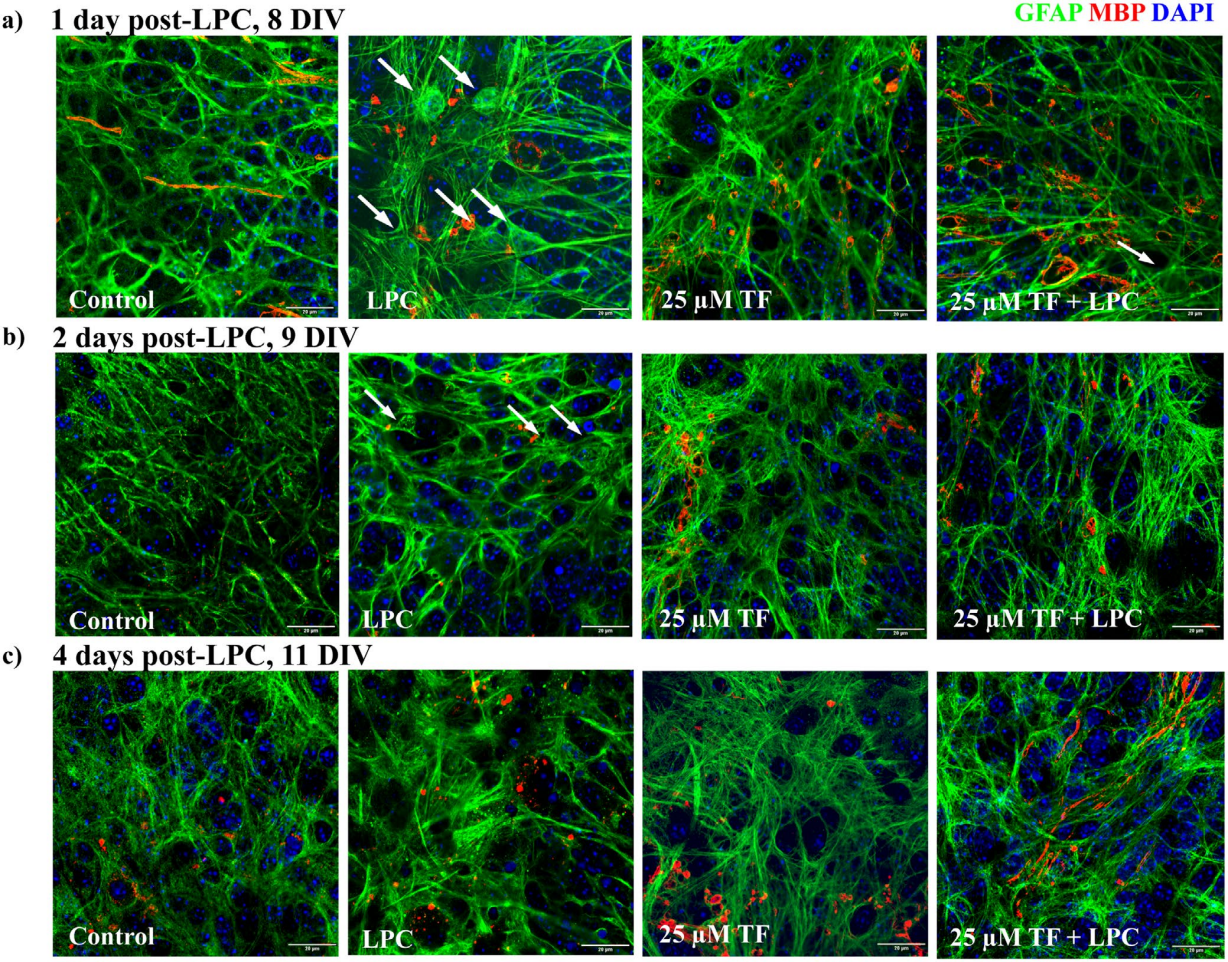
Morphological alterations of astrocytes following teriflunomide administration during demyelination. OSC were treated with 25 μM teriflunomide starting at 6 DIV until fixation. Representative images of GFAP stained slices at 1 day (a) (8 DIV), 2 days (b) (9 DIV), and 4 days (c) (11 DIV) after LPC treatment. Arrows indicate morphological changes of astrocytes, which were attenuated after treatment with 25 μM teriflunomide. Scale bar: 20 μM. DIV, days‐in vitro; LPC, lysolecithin; TF, teriflunomide.

In order to evaluate the effects of teriflunomide on glial cell reactions during demyelination, astrocytes and microglia were immunohistochemically stained using the microglial marker IBA‐1 and the astroglial marker GFAP, respectively. Immunofluorescent staining for GFAP revealed pronounced morphological alterations of astrocytes in the cerebellar white matter following LPC treatment (Figure 6). Astrocytes in LPC‐treated slices displayed hypertrophic cell bodies and thickened processes compared to control conditions. In slices treated with teriflunomide in the presence of LPC, astrocytes exhibited a morphology more comparable to that observed in control slices, characterized by less pronounced cellular hypertrophy and thinner processes. As these observations were based on morphological inspection of GFAP immunoreactivity, no quantitative assessment of astrocyte activation or astrocyte density was performed.

**FIGURE 7:**
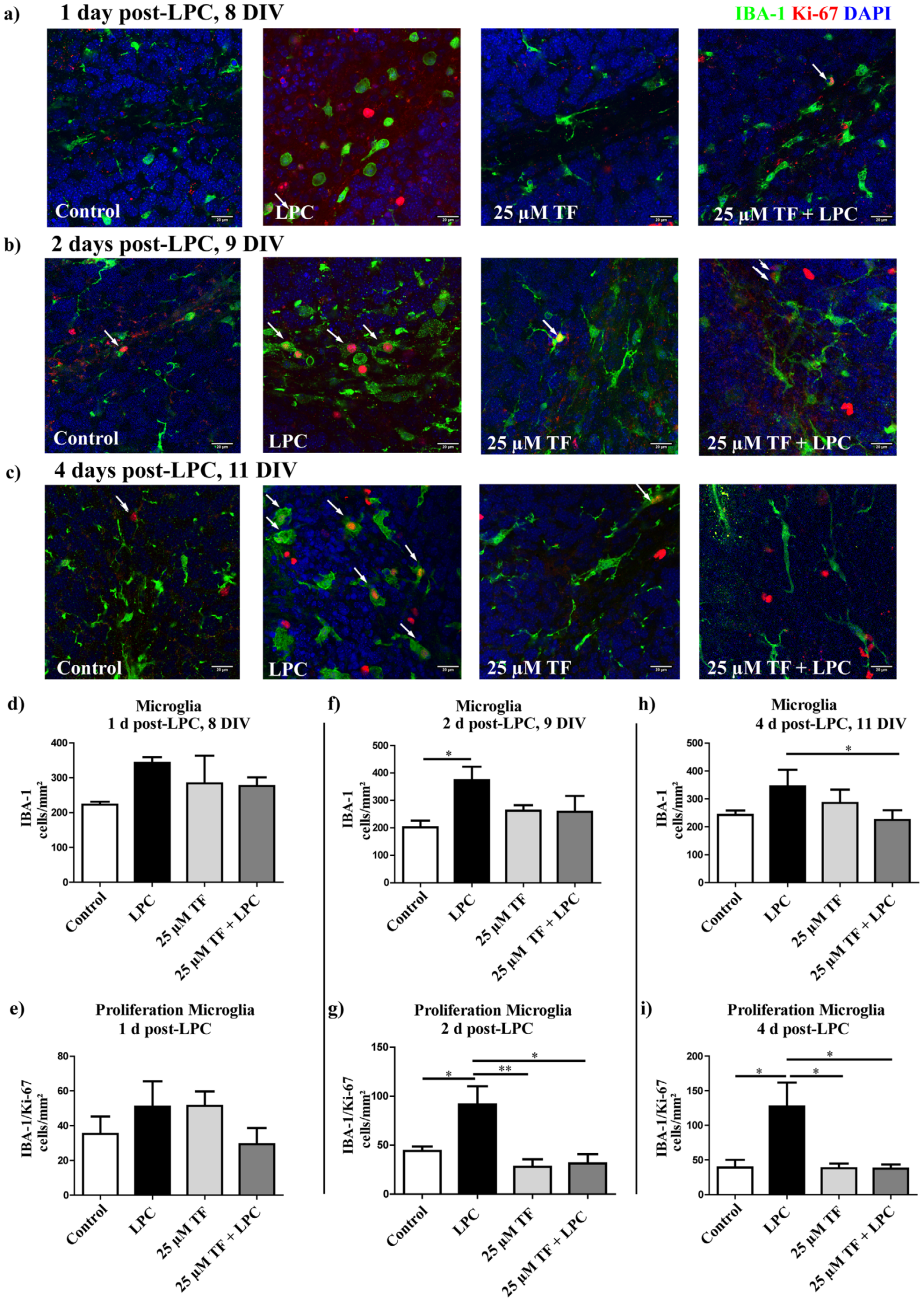
Effects of teriflunomide on microglia proliferation in OSC. Cell densiteis and proliferation of microglia was determined by IBA‐1 and Ki‐67 double staining. OSC were treated with 25 μM teriflunomide from the Day 6 DIV, and slices were fixed at one (a) (8 DIV), two (b) (9 DIV), and four (c) (11 DIV) days LPC. In d, f, and h the graphs show the number of IBA‐1 positive cells (microglia) in the withe matter. Graphs in (e, g, and i) depicture density of proliferating microglia during demyelination in OSC. The simultaneous treatment with 25 μM teriflunomide could abolish an increase of microglial proliferation caused by LPC. Data are represented as mean ± SEM (*n* = 4–8). Statistical analysis was performed using Kruskal–Wallis test followed by Dunns post hoc tests (*p < 0.05). Arrows indicate proliferation of microglia indicated by double positive cells. Scale bar 20 μM. DIV, days‐in vitro; LPC, lysolecithin; TF, teriflunomide.

The proliferation of microglia was determined by IBA‐1 and Ki‐67 double staining. Two days post‐LPC, during severe demyelination (9 DIV), the total numbers of microglia as well as the amount of proliferating microglia were significantly increased (Figure 7f,g). The number of proliferating microglia remained significantly elevated following LPC‐induced demyelination, even after two further days (11 DIV) of exposure (Figure 7i). However, the simultaneous treatment with 25 μM teriflunomide significantly attenuated the LPC‐induced increase in total microglial cell numbers as well as microglial proliferation, resulting in microglial cell densities comparable to untreated control slices (both, at 9 and 11 DIV), indicating an important role of teriflunomide in regulating microglia activity during demyelination (Figure 7). In addition to changes in microglial cell density and proliferation, pronounced alterations in microglial morphology were observed across experimental conditions. Under control conditions, IBA‐1‐positive microglia displayed a ramified morphology with elongated processes and small cell bodies, consistent with a surveillant morphological state. LPC treatment induced a marked shift toward a more rounded, amoeboid morphology characterized by enlarged cell bodies and reduced process complexity, indicative of a pronounced morphological remodeling of the microglial population during acute demyelination. In slices treated with teriflunomide alone, microglia largely retained a ramified morphology similar to controls. Notably, combined LPC and teriflunomide treatment resulted in a distinct intermediate morphology, characterized by elongated but thickened processes and enlarged somata, which differed clearly from both LPC‐only and control conditions.

**FIGURE 8:**
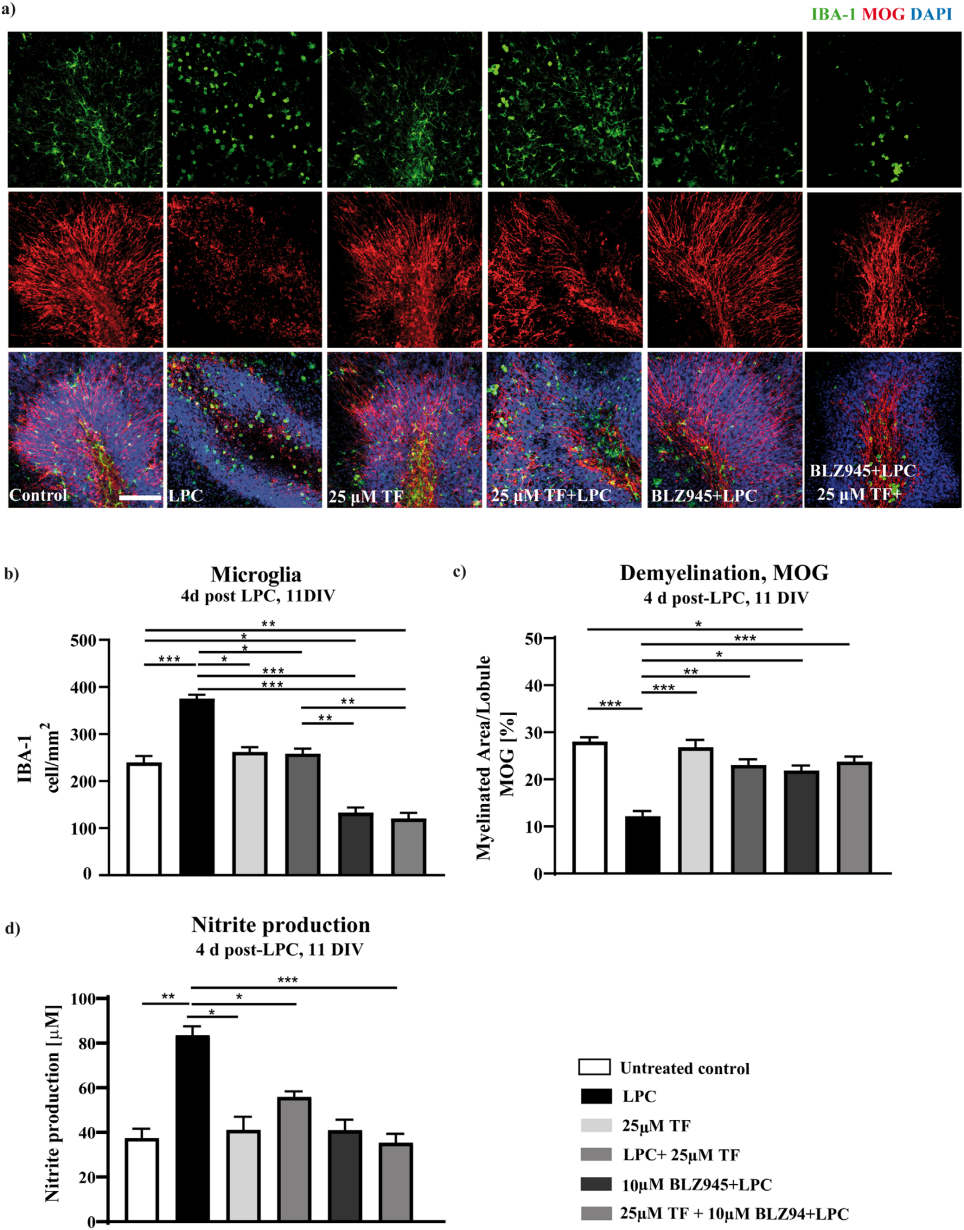
Effects of microglia depletion on myelin preservation during demyelination. The possible relation between myelin preservation (MOG) and microglia population (IBA‐1) was investigated during demyelination by IBA‐1 and MOG double staining (a). OSC were treated with 25 μM teriflunomide from the Day 6 DIV and slices were fixed at 4 (11 DIV) days post‐LPC. Simultaneous treatment with 25 μM teriflunomide and the CSF‐1R inhibitor BLZ945 reduced LPC‐induced elevation of microglia numbers along with myelin degradation. Quantification of MOG density (c), numbers of IBA‐1+ microglia (b), and nitrite production in BLZ945‐treated cultures (d). Data are represented as mean ± SEM (*n* = 14–17). Statistical analysis was performed using Kruskal–Wallis test followed by Dunns post hoc tests (*p < 0.05; **p < 0.01; ***p < 0.001). Arrows indicate proliferation of microglia indicated by double positive cells. Scale bar 100 μM. DIV, days‐in vitro; LPC, lysolecithin; TF, teriflunomide.

Concomitantly, nitrite concentrations in the culture medium were significantly increased following LPC treatment. This increase was reduced in slices simultaneously treated with LPC and teriflunomide and closely reflected the differences observed in microglial cell density between experimental groups. Of note, nitrite levels correlate with microglial cell density (Figure 8d). To further investigate whether reduced microglial abundance is associated with preserved myelin integrity during demyelination, OSC were treated with the colony‐stimulating factor 1 receptor (CSF‐1R) inhibitor BLZ945, also known as sotuletinib, to partially deplete microglia. Sotuletinib depletes especially inflammatory microglia because CSF‐1R is critical for their survival [20]. BLZ945 treatment resulted in a marked reduction in the number of IBA‐1‐positive microglial cells during the demyelination phase (Figure 8a,b), highlighting the importance of microglia and their modulation in the OSC model. Similarly, the slices treated with teriflunomide upon LPC induced demyelination produced comparable nitrite levels to those depleted of microglia by BLZ945 treatment, further suggesting a correlation to reduced microglial cell numbers (Figure 8d).

Concomitantly, MOG immunoreactivity was preserved in BLZ945‐treated slices compared to LPC‐treated controls (Figure 8c). The extent of MOG preservation observed after BLZ945 treatment was comparable to that seen following teriflunomide administration, indicating that reduced microglial abundance is associated with diminished myelin loss in the LPC‐induced demyelination model.

### 3.5 Teriflunomide Exerts Positive Effects on Oligodendrocytes and Myelin Protein Expression During Remyelination

**FIGURE 9:**
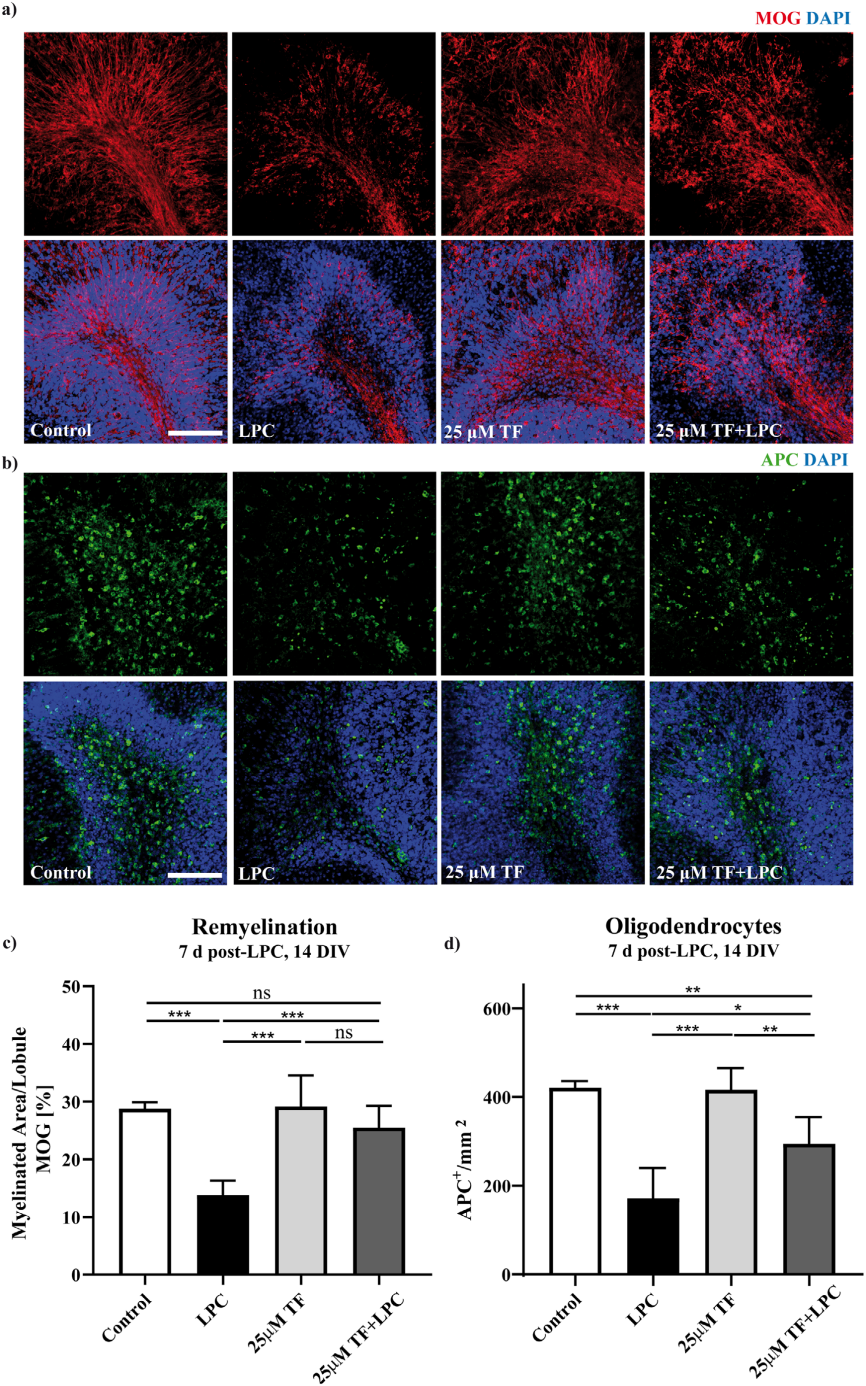
Effect of teriflunomide on remyelination and oligodendrocytes in OSC. To study remyelination cultures were treated with 25 μM of teriflunomide from 9 DIV until 14 DIV (see Figure 1). Quantification of MOG showed positive effects of 25 μM teriflunomide on remyelination (a, c). Representative images of slices at 7 days (14 DIV) post‐LPC treatment, stained against MOG, are shown in (a). Representative images of slices at 7 days (14 DIV) post‐ LPC treatment, stained with APC antibodies (mature oligodendrocytes) are shown in (b). Positive effect of teriflunomide on mature oligodendrocytes during remyelination is shown in (d). Data are represented as mean ± SEM (*n* = 17–25). Statistical analysis was performed using Kruskal–Wallis test followed by Dunns post hoc tests. Scale bar 100 μm. DIV, days‐in vitro; LPC, lysolecithin; TF, teriflunomide.

To assess the effect of teriflunomide on remyelination of OSC, slices were incubated with 25 μM teriflunomide from Day 2 post‐LPC (9 DIV) until at seven (14 DIV) days post‐LPC. Quantification of spontaneous remyelination after LPC showed an increased relative density of myelinated segments (stained with MOG) in the presence of teriflunomide compared to non‐treated cultures during remyelination (Figure 9a,c). However, whereas the density of MOG is statistically no longer different from untreated control slices, the fine structure of myelin was not yet fully restored (Figure 9a,c). At the same time, the number of oligodendrocytes increases significantly under teriflunomide treatment (Figure 9b,d).

### 3.6 Teriflunomide Reduces Cell Numbers in Isolated OPC Cultures

**FIGURE 10:**
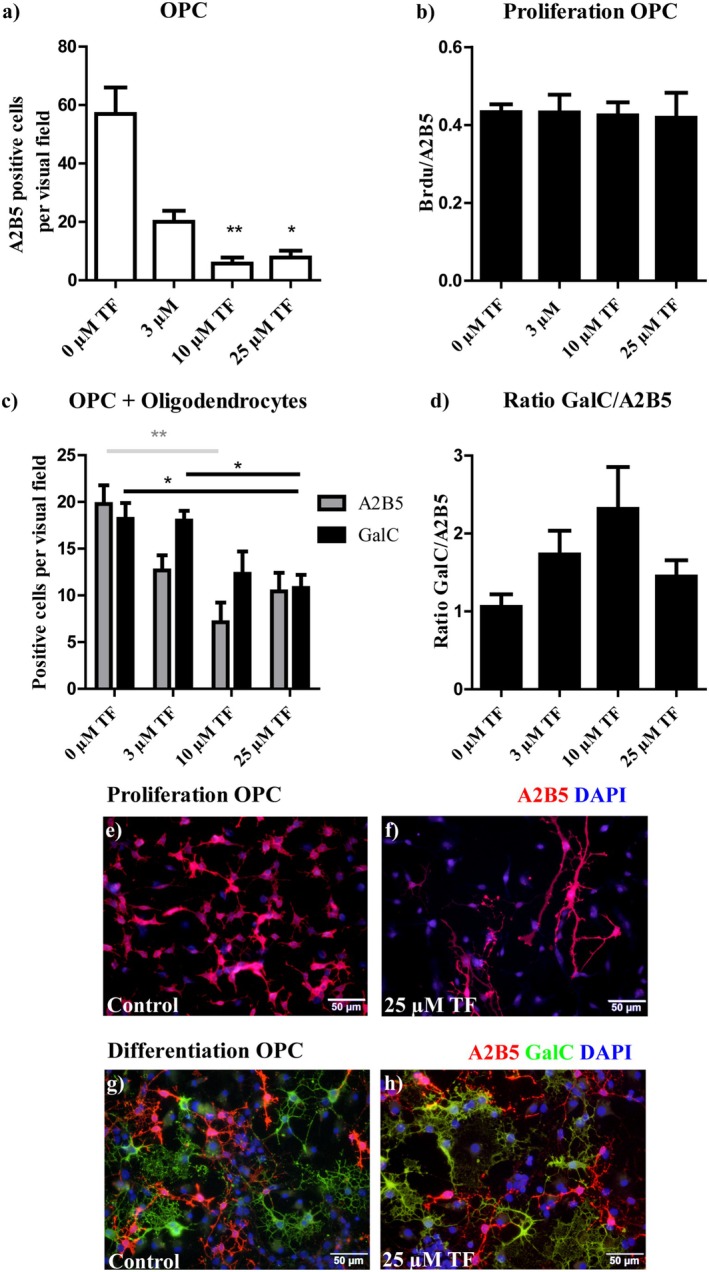
Effects of teriflunomide on primary oligodendrocytes. Primary OPC were treated with 3, 10, or 25 μM teriflunomide for 48 h. Proliferation of OPC in proliferation medium (a, total numbers of A2B5+ cells; b, ratio of BrdU/A2B5+/cells; e and f, representative pictures of A2B5 positive cells in proliferation medium) and differentiation of OPC in differentiation medium (c, total numbers of GalC+ cells and A2B5+ OPC; d, ratio of GalC+ cells to A2B5+ OPC; g and h, representative pictures of A2B5/GalC stained cells) were determined by immunohistochemical staining. Data are presented as mean ± SEM (*n* = 5–10). Statistical analysis was performed using Kruskal–Wallis test followed by Dunns post hoc tests. (*p < 0.05; **p < 0.01; ***p < 0.001). Scale bar 50 μm. TF, teriflunomide.

Since teriflunomide prevented demyelination in OSC and positively impacted the number of mature oligodendrocytes during remyelination in OSC, we aimed to elucidate possible direct effects of teriflunomide on oligodendrocytes. Therefore, 3, 10, or 25 μM teriflunomide was added for 48 h to oligodendrocyte cultured in proliferation medium or differentiation, respectively. As shown in Figure 10b the proliferation index calculated by the ratio of BrdU/A2B5 double‐positive cells was not changed after teriflunomide treatment, but absolute numbers of A2B5 positive cells decreased significantly after an incubation with 10 or 25 μM teriflunomide (Figure 10a,e,f). Similarly, in the differentiation medium, the total number of both A2B5 and GalC positive cells decreased after teriflunomide treatment, suggesting even toxic effects of teriflunomide on isolated OPC especially at higher teriflunomide concentrations (Figure 10c,d,g,h).

### 3.7 Astrocyte Supernatants Prevent the Decline of OPC After Teriflunomide Treatment

**FIGURE 11:**
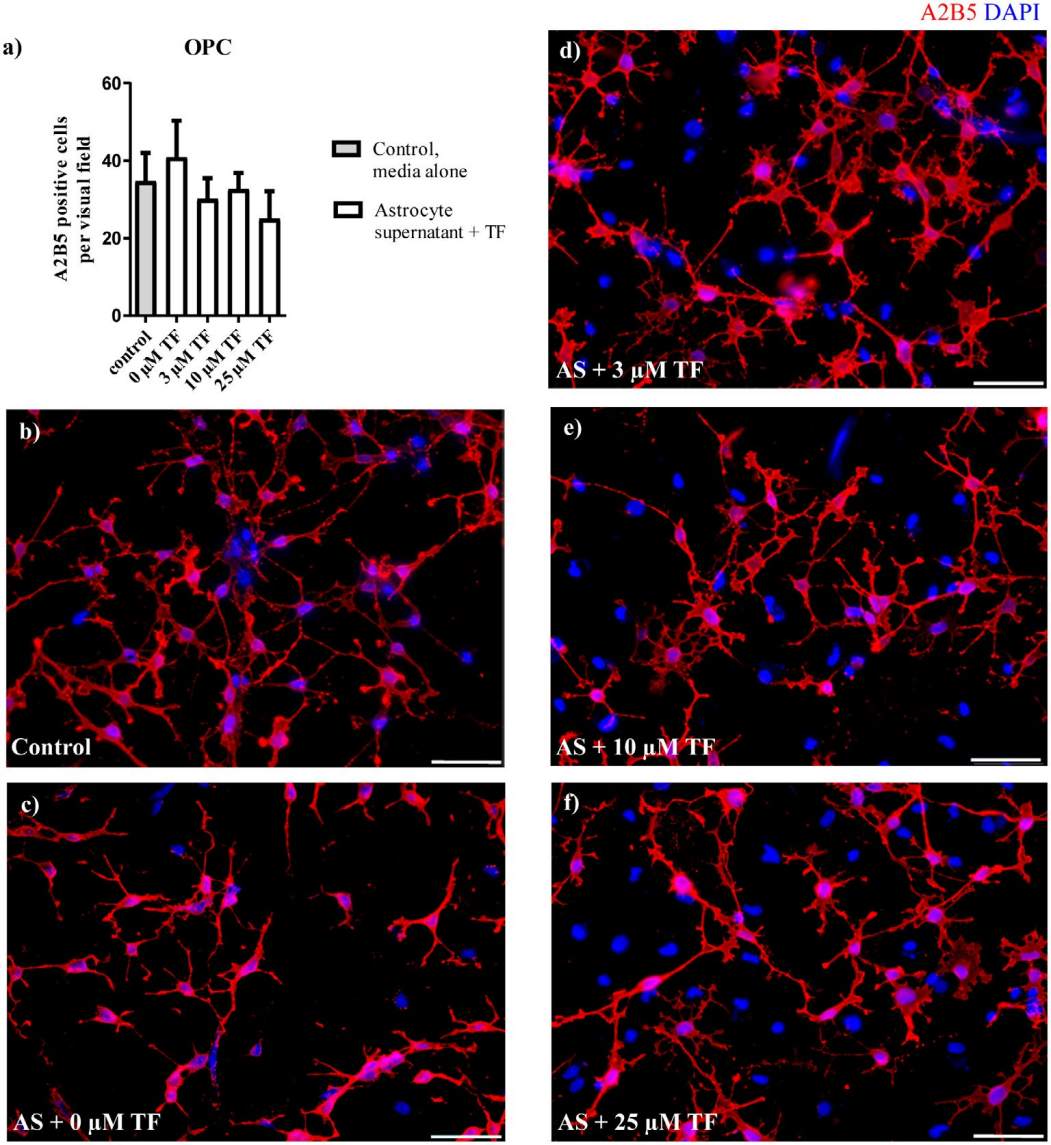
Astrocyte supernatants prevent the decline of OPC after teriflunomide treatment. (a) Absolute numbers of A2B5+ OPC were determined after incubation of oligodendroglial cultures with 3, 10, or 25 μM teriflunomide supplemented with supernatants (medium) from unstimulated astrocytes. (b–f) Representative pictures show A2B5 stained oligodendroglial cultures in proliferation medium supplemented with astrocyte supernatants and stimulated with different concentrations of teriflunomide. Data are represented as mean ± SEM (*n* = 5). Statistical analysis was performed using Kruskal–Wallis test followed by Dunns post hoc tests. Scale bar 50 μm. AS, astrocyte supernatant; TF, teriflunomide.

As we observed a decline of A2B5 positive OPC after direct treatment of cells with 3, 10, or 25 μM teriflunomide, we next aimed to elucidate if astrocytic factors could abolish this effect. Indeed, after addition of supernatants of untreated astrocytes to OPC cultures treated with teriflunomide in vitro, the number of A2B5‐positive oligodendrocytes did not change significantly compared with control cultures (Figure 11) indicating a survival promoting effect of astrocyte‐derived factors on OPC. Hence, we hypothesized that in the OSC cultures, resident astrocytes might prevent the OPC/oligodendrocytes‐diminishing effect of teriflunomide administration, which was observed in isolated oligodendrocyte cultures in vitro.

### 3.8 Supernatants From Teriflunomide Treated Microglia Exert no Effects on Oligodendrocyte Cultures

**FIGURE 12:**
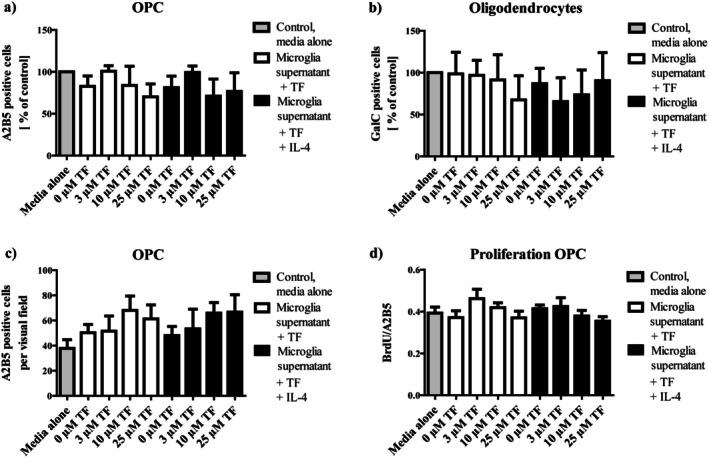
Differentiation and proliferation of oligodendrocytes treated with teriflunomide and microglia supernatant. Numbers of A2B5^+^ OPC (a) and of GalC^+^ cells (b) were determined after incubation of oligodendroglial cultures in differentiation medium supplemented with supernatants from microglia treated with 3, 10, or 25 μM teriflunomide (with/without IL‐4). Proliferation of A2B5^+^ OPC (c) and proliferation index (d) were determined after incubation of oligodendroglial cultures in proliferation medium supplemented with supernatants from microglia treated with 3, 10, or 25 μM teriflunomide. Data are represented as mean ± SEM (*n* = 4–8). Statistical analysis was performed using Kruskal–Wallis test followed by Dunns post hoc tests. TF, teriflunomide.

To determine if microglia treated with teriflunomide influence differentiation or proliferation of OPC, we cultured OPC in proliferation or differentiation media supplemented with pretreated microglia supernatants. These experiments were designed to test potential indirect effects of teriflunomide‐treated microglia on OPC behavior, despite the absence of direct effects of teriflunomide alone. Absolute numbers of A2B5 (immature OPC) and GalC (mature oligodendrocytes) positive cells remained unchanged after incubation of OPC in the differentiation medium supplemented with supernatants from teriflunomide‐treated microglia as compared to the untreated control (Figure 12a,b). Proliferation was determined by calculating the BrdU/A2B5 double‐positive cells ratio after the incubation of OPC in proliferation medium supplemented with supernatants from teriflunomide‐treated microglia. Proliferation index as well as total numbers of A2B5 positive progenitors remained unchanged (Figure 12c,d). Similarly, we did not observe any effect of M2 derived microglia (microglia were pretreated with teriflunomide plus IL‐4) supernatants on proliferation or differentiation of OPC.

## 4 Discussion

The aim of this study was to investigate the effects of teriflunomide on glial cells during myelination, demyelination, and remyelination in OSC, since previous animal studies have shown that teriflunomide can cross the blood–brain barrier and modulate the functions of glial cells [7, 8, 21, 22]. Here, using the LPC‐induced demyelination OSC model, we demonstrate that teriflunomide significantly improved the amount of compact myelin, as shown by MOG staining, and increases the number of mature APC+ oligodendrocytes during remyelination. Previously Martin and colleagues have already shown that teriflunomide promoted the differentiation of newly generated oligodendrocytes and significantly increased remyelination in the demyelinated spinal cord of mice treated with LPC [22]. Another study by Gottle et al. [8] demonstrated that orally administrated teriflunomide positively affects the remyelination‐related oligodendroglial dynamics both after pulsed and constant application in vivo during a 6‐week period of continuous cuprizone feeding. A few years earlier, Gottle et al. [9] have shown that teriflunomide (5 μM) may enhance OPC differentiation in vitro. However, this was only the case in an experimental setting with pulsed treatment. In addition, Martin et al. [22] applied teriflunomide at very low concentrations in their in vitro experiments (10 nM) observed a significant increase in differentiating oligodendrocytes as well. Interestingly, Lycke et al. [21] found in their recent pharmokinetic study that the mean terifunomide concentrations in plasma and CSF of teriflunomide (14 mg daily) treated patients with RRMS (without relapse or disability worsening within 6 months) were 38,775 (SEM ±7256) ng/mL (143 μM) and 68 (SEM ±15) ng/mL (0.25 μM), respectively and the passage of terifunomide over the blood‐cerebrospinal fluid barrier (BCSFB) was less than 1% [21]. However, in naïve and EAE‐exposed rats, the concentration in the CNS after a single oral dose of [14C]‐terifunomide reaches concentrations of 2.5–4.1 μM (676–1108 ng/mL) [7]. This exceeds the IC50 values for inhibition of DHODH by teriflunomide (270 ng/mL, 1 μM) [23], suggesting that teriflunomide may exert some effects on neuronal and glial cells in the CNS. In our in vitro studies, we did not observe any proliferative effect of teriflunomide, nor could we recapitulate the pro‐differentiating effect of teriflunomide on purified OPC, using 3–25 μM teriflunomide continuously, what is probably closer to the physiological situation where the drug is administrated to patients with MS on a long‐term basis. Now it appears very plausible that the response of the oligodendrocytes is extremely sensitive to the teriflunomide dosage and that low concentrations are more favorable for the isolated oligodendrocytes. In our in vitro experiments using 3–25 μM teriflunomide, we observed a slight increase in the differentiation index (ratio A2B5/CalC+ cells) after teriflunomide treatment. However, this effect was probably more due to an increased mortality rate of the undifferentiated A2B5 positive cells observed upon teriflunomide treatment (3–25 μM) in both proliferation‐ but also in differentiation‐evoking conditions. Gottle et al. [9] showed that apoptotic oligodendrocytes were increased even by a low concentration of 1 μM teriflunomide, suggesting that this impaired cell survival is caused by induction of pyrimidine stress. In our experiments, this negative impact of high dosage of teriflunomide was abolished after addition of supernatants from astrocytes, implying that these effects are probably ameliorated in OSC or in vivo with a constant astrocytic support. In organotypic slice cultures, OPC are embedded within a complex cellular environment and are supported by astrocytes, microglia, and extracellular matrix components, which provide trophic and metabolic support. Under these conditions, treatment with 25 μM teriflunomide did not significantly alter the basal density of proliferating OPC. In contrast, isolated OPC cultures lack this supportive cellular milieu and therefore represent a more vulnerable system. Accordingly, teriflunomide treatment resulted in a pronounced reduction in A2B5‐positive OPC density in vitro. These findings indicate that the effects of teriflunomide on OPC survival or proliferation are strongly context‐dependent and can be modulated by glial cell–derived support signals present in organotypic slice cultures.

Furthermore, we show here that teriflunomide has a myelin protective effect and LPC induced demyelination is significantly diminished after incubation with 25 μM; however, concentrations of 3 and 10 μM did not exert this effect. Furthermore, ultrastructural quantification transmission electron microscopy supported the immunohistochemical findings by demonstrating preservation of myelinated axons in teriflunomide‐treated slices. In addition, exploratory g‐ratio analysis revealed significant preservation of intact myelin after application of teriflunomide in this demyelination model. The mechanisms of myelin protection are not yet clear and may involve effects via microglia and/or astrocytes.

The role of polarized microglia during de‐ and remyelination is not fully understood, but it has been shown that activated microglia clear myelin debris by enhanced phagocytosis and release of cytokines as well as growth factors that may stimulate OPC differentiation [24]. Furthermore, polarized microglia can act as immunomodulators to drive remyelination in the OSC model [15]. On the other hand, microglia can also release cytotoxins and pro‐inflammatory factors which contribute to oligodendrocyte damage [25]. In the LPC‐induced demyelination model used in this study, a pronounced increase in microglial cell numbers and proliferation was observed during the acute demyelination phase. Treatment with teriflunomide consistently attenuated this LPC‐induced expansion of the microglial population. Similar results were obtained in a former study where a lower teriflunomide concentration of 5 μM reduced the proliferation of microglia in mixed glial cell cultures [10]. Furthermore, microglial density was decreased upon teriflunomide treatment in both a Theiler's murine encephalomyelitis virus (TMEV) mouse model and a mouse model of traumatic brain injury, indicating reduced inflammation [26, 27]. Additionally, in a mouse model of transient middle cerebral artery occlusion (tMCAO), mice treated with teriflunomide showed reduced numbers of activated IBA‐1‐positive microglia and lower protein levels of interleukin‐1β (IL‐1β), cyclooxygenase‐2 (COX‐2), and 3‐Nitrotyrosine (3‐NT) [28].

The observed changes in microglial morphology across experimental conditions indicate pronounced microglial dynamics in the organotypic slice culture model. However, morphological alterations alone do not allow conclusions regarding specific activation states or inflammatory polarization. Generally amoeboid microglial morphologies have been associated with phagocytic and inflammatory responses in various experimental settings, however, such associations remain context‐dependent and require further confirmation by molecular or functional markers. In the present study, microglial morphology changes are therefore interpreted as a structural correlate of microglial population remodeling rather than as a definitive indicator of activation or inflammatory phenotype. Increased mRNA expression of inflammatory‐related molecules, such as TNF‐a, CCl 3, IL‐6, CxCl 10, and osteopontin upon demyelination was revealed by our initial experiments using OSC culture as well as in our recent publication [29]. Nevertheless, these experiments were conducted by examining the expression of mRNA in entire tissues, which precluded the capacity to ascertain the specific cell population responsible to produce these factors.

Partial depletion of microglia using the CSF‐1R inhibitor BLZ945 resulted in preservation of MOG immunoreactivity during LPC‐induced demyelination. This finding closely mirrors the effects observed after teriflunomide treatment and supports the concept that microglial abundance is functionally linked to the extent of myelin loss in this ex vivo model. While BLZ945 treatment does not allow conclusions regarding microglial activation states or specific effector mechanisms, the convergence of teriflunomide‐ and BLZ945‐mediated effects strongly suggests that limiting microglial expansion during acute demyelination is sufficient to attenuate myelin degradation. Therefore, there might also exist a microglia‐specific effect of teriflunomide in myelin preservation and prevention of brain damage after LPC in OSC by modulating the microglia phenotype. Interestingly, Wolters and colleagues [30] observed attenuation of brain injury and cell death after administration of teriflunomide under ischemia‐like conditions of oxygen–glucose deprivation in cerebellar slices.

Importantly, the present study primarily assessed microglial abundance and proliferation rather than microglial activation states or inflammatory phenotypes. Although increased nitrite concentrations were detected following LPC treatment, these levels closely followed changes in microglial cell density and therefore likely reflect the increased number of microglia rather than enhanced activation on a per‐cell basis. Consequently, no direct conclusions can be drawn regarding a potential shift toward pro‐ or anti‐inflammatory microglial phenotypes in response to teriflunomide treatment. Assessment of microglial activation markers or cytokine profiles would be required to address this question and represents a limitation of the current study. Nevertheless, the observation that partial depletion of microglia using the CSF‐1R inhibitor BLZ945 similarly preserved myelin integrity strongly supports a functional association between microglial abundance and the extent of LPC‐induced demyelination in this ex vivo model. These findings suggest that limiting microglial expansion during acute demyelination may contribute to myelin preservation, independent of specific microglial activation states.

Indirect effects of teriflunomide pretreated microglia were also tested in primary oligodendrocyte cultures in vitro. Supernatants derived from teriflunomide pretreated microglia did not alter proliferation or differentiation of OPC. IL‐4 treatment was employed as an established approach to induce anti‐inflammatory or alternatively activated priming of microglia. However, IL‐4 stimulation alone does not permit conclusions regarding complete or uniform polarization of microglia into a defined M2 phenotype. In the present study, no molecular markers of microglial polarization, such as Arginase‐1 or iNOS, were assessed. Accordingly, the IL‐4 experiments are interpreted as exploratory and contextual, aiming to modulate the inflammatory milieu rather than to define specific microglial activation states. Previous work has shown that LPC‐induced demyelination is associated with alterations in microglial arginine metabolism and loss of Arg1 expression [15, 29], underscoring the relevance of inflammatory context in this model. Nevertheless, direct assessment of microglial polarization states was beyond the scope of the present study.

In addition, effects of teriflunomide on astrocytes are reported as well [31]. Kabiraj et al. [31] have recently shown that reconditioning with teriflunomide prevented TNFα‐induced shifts in oxidative phosphorylation, decreased mitochondrial ATP production, and reduced astrocytic inflammatory responses, suggesting that this drug may limit neuroinflammation through its action as a metabolic modulator. Therefore, we investigated if teriflunomide could affect astrocyte morphology by GFAP immunostainings. The morphology was altered after LPC treatment, but not after simultaneous treatment with teriflunomide. This LPC‐induced astrocyte swelling might be similar to the phenotype that is observed in different models of brain injuries [32]. Thus, teriflunomide treatment of OSC seemed to result in less activated astrocytes. However, although GFAP immunostaining revealed pronounced morphological alterations of astrocytes following LPC treatment, these observations were qualitative in nature. No quantitative parameters of astrocyte activation or astrocyte density were predefined in the experimental design. Consequently, the present study does not allow conclusions regarding astrocytic activation states or functional phenotypes. Future studies employing quantitative morphometric analyses or molecular markers will be required to further characterize astrocyte responses in this model. The observation that astrocyte‐conditioned medium rescued the teriflunomide‐induced reduction in OPC numbers suggests the presence of astrocyte‐derived protective or supportive factor(s). However, the identity of these factor(s) and their regulation by teriflunomide were not addressed in the present study. Importantly, astrocyte‐conditioned media were generated from untreated astrocytes, and we did not assess whether teriflunomide alters the production or release of astrocyte‐derived mediators. Therefore, no conclusions can be drawn regarding potential effects of teriflunomide on astrocyte‐secreted protective factors. Elucidating the molecular nature of these astrocyte‐derived signals and their regulation by teriflunomide represents an important direction for future investigations.

In summary, the evidence from the literature and our results suggests that teriflunomide exerts a positive beneficial effect on the CNS during pathological events by attenuating microgliosis, modulating astrocyte phenotype, promoting OPC differentiation and myelination, which may be, however, modulated by the concentration of teriflunomide or the duration of teriflunomide treatment [33]. On the other hand, it is also theoretically possible that microglia and astrocytes have a lower activation potential due to the less severe damage to OPC/myelin, resulting in a reduced accumulation of debris, which should have a purely indirect effect on these cells via teriflunomide.

## 5 Conclusions

In this study, we demonstrate that teriflunomide attenuates myelin degradation during LPC‐induced demyelination and enhances spontaneous remyelination in organotypic cerebellar slice cultures. Teriflunomide treatment was consistently associated with a reduction in microglial cell density and proliferation during the acute demyelination phase. Partial depletion of microglia using the CSF‐1R inhibitor BLZ945 resulted in a comparable preservation of myelin, supporting a functional association between microglial abundance and the extent of myelin loss in this ex vivo model. Quantitative ultrastructural analyses further supported the presence of preserved compact myelin structures in teriflunomide‐treated slices. While the present study does not allow conclusions regarding specific microglial activation states or inflammatory phenotypes, the data suggest that limiting microglial expansion during demyelination is associated with reduced myelin damage. In addition, teriflunomide promoted remyelination and increased the number of mature oligodendrocytes in the recovery phase, without exerting direct pro‐proliferative effects on isolated oligodendrocyte progenitor cells in vitro. Together, these findings indicate that teriflunomide exerts beneficial effects on myelin integrity and remyelination in an ex vivo demyelination model, potentially through modulation of glial cell dynamics rather than direct effects on oligodendroglial cells. Further studies will be required to elucidate the underlying molecular mechanisms and to define the contribution of specific microglial functional states.

## Author Contributions

Conceptualisation: M.S., V.G. Design of experiments: M.S., V.G., J.K., W.B. Performance of experiments: J.K. L.‐J.S. H.R., S.H. Analysis of experiments: M.S., V.G., W.B. F.H. J.K. L.‐J.S. Preparation of original draft: J.K., V.G. Review and editing: M.S., V.G., L.‐J.S., W.B., F.H, S.T. Funding acquisition: M.S. All authors read and approved the final manuscript.

## Funding

The study was partly supported by the Sanofi (10.13039/100004339).

## Ethics Statement

Not applicable for humans since there are no human subjects or samples in this study. All procedures involving animals were approved by the Local Institutional Animal Care and Research Advisory Committee and permitted by the local authority (Lower Saxony State Office for Consumer Protection, Food Safety, and Animal Welfare Service) following the legal rules (German Animal Welfare Law, TierSchG, §4, Abs. 3) for sacrifice of animals for research purposes (MHH institutional registry numbers: §4/2016/112 and §4/2018/193).

## Consent

The authors have nothing to report.

## Conflicts of Interest

The authors declare no conflicts of interest.

## Data Availability

The data used in this study are available from the corresponding authors upon reasonable request.
